# Determinants of breast cancer among women attending oncology units in selected health facilities of Hawassa City, Sidama Region, Southern Ethiopia, 2023: case-control study

**DOI:** 10.3389/fonc.2024.1352191

**Published:** 2024-04-12

**Authors:** Selamawit Kebede, Tsegaye Alemu, Ashenafi Mekonnen

**Affiliations:** ^1^ Public Health Department, Yanet-Liyana College of Health Sciences, Hawassa, Ethiopia; ^2^ School of Public Health, College of Medicine and Health Sciences, Hawassa University, Hawassa, Ethiopia

**Keywords:** breast cancer, case–control, determinates, oncology units, Sidama Region Ethiopia

## Abstract

**Background:**

The incidence of breast cancer (BC) is rampantly increasing in developing countries particularly Ethiopia. Unfortunately, the morbidity and mortality rates are sharply increasing, and because of this, families are suffering from socioeconomic crises. Despite this, there is limited evidence on the determinants of breast cancer in Ethiopia as well as in the study area.

**Objective:**

To identify the determinants of BC among women attending oncology units in selected hospitals in Hawassa City, Ethiopia, in 2023.

**Method:**

A hospital-based, case-control study with 300 patients (75 cases and 225 controls) was carried out in Hawassa from June to July 2023. A simple random sampling technique was used to select cases and controls. Data were collected via pretested and structured digitally installed questionnaires with Kobo collection/smartphones. The data were exported from the server to SPSS version 27 for analysis. Descriptive analysis of univariate, bivariate, and multivariable logistic regression data was conducted to determine the associations between breast cancer incidence and independent factors.

**Results:**

A total of 300 women participated in this study for a response rate of 100%. The mean ( ± SD) ages of the respondents were 37.2 ( ± 14.8) and 36.6 ( ± 15.1) years for the cases and controls, respectively. According to the multivariate logistic regression model, postmenopausal status [AOR: 2.49; 95% CI (1.18, 5.23)], family history of cancer [AOR: 2.33; 95% CI (1.12, 4.82)], oral contraceptives [AOR: 2.74; 95% CI (1.34, 5.99)], overweight and/or obesity [AOR: 2.29; 95% CI: (1.14, 4.59)], and consumption of solid oil [AOR: 2.36; 95% CI (1.20, 4.67)] were independently associated with BC risk.

**Conclusion:**

This study revealed important risk factors for BC. Therefore, women should adopt healthier lifestyles through healthy nutrition and regular exercise to reduce the risk of developing BC. In addition, early detection and regular screening are proactive approaches for detecting BC.

## 1 Introduction

Breast cancer (BC) is the most prevalent cancer in women globally accounting for 2.3 million new cases and one in six cancer deaths in 2020 (1). Globalization has led to a greater incidence of BC in high-income countries (571/100,000) than in low-income countries (95/10,000) (2). Due to globalization, risk factors for cancer are appearing with our modern diet, which emphasizes super grain and more processed food; the use of addictive substances, toxic and pharmaceutical products; and waste exposure. However, the prevention of cancer still depends on the recognition and elimination of risks from carcinogens (3). The five most prevalent nations were all in Europe. The number of new cases in Africa is estimated to have been 92,600 in 2008 and 133,900 in 2012 (1). In 2020, 2.3 million women were diagnosed with BC, and 685,000 women died from BC worldwide (4, 5).

In Africa, 8% of all BC cases are diagnosed, but the mortality rate is far higher than expected (1). Moreover, sub-Saharan Africa has the highest BC incidence and mortality rates attributed to Westernized lifestyles, food changes, and reduced physical activity among the African population (6). In 2020, there was significant geographical variation in the major cancers in sub-Saharan Africa (7).

In Ethiopia, BC is the most prevalent cancer accounting for 17.7% of cancer deaths and 22.6% of all cancer cases annually (8, 9). In addition, every year, approximately 60,000 new cases of BC are diagnosed. Similarly, evidence from the Addis Ababa cancer registry report shows that BC is responsible for 23% of all cancer cases and 33% of all cancer cases in women (10). Moreover, it is the most common malignancy in Ethiopia with increased rates of mortality and morbidity. For instance, of the 4,139 new cancer patients diagnosed between 2012 and 2013, 67% were female, and 31.5% had BC. The age-standardized incidence rate of BC was 43.3 per 100,000 (11) females, and a yearly average of 216 incident cases of BC were reported between 1997 and 2012 (11).

Multiple factors, such as alcohol consumption, tobacco use, obesity, inactivity, diet, family history, early menarche, late menopause, late age, null parity, and nonbreastfeeding practices, are known to increase the risk of BC (8). In contrast, lifestyle modifications, targeted prevention programs, and population-based screening can significantly reduce BC incidence (12).

Unfortunately, even if all of the potentially modifiable risk factors could be mitigated, this would only reduce the risk of developing BC by at most 30% (13). In addition, 20%–30% o BCs can be ascribed to controllable characteristics, and 5%–10% of BCs can be related to factors such as genetic mutations and family history (14). Thus, the healthcare system should be strengthened, and the gradual adoption of universal health coverage should be encouraged especially for low- and middle-income nations where cancer and noncommunicable disease programs are frequently inaccessible and limited-resource citizens are critical (10).

The World Health Organization launched the Global BC Initiative to address the increasing burden of BC, which is estimated to kill 685,000 women globally in 2020 (1). Similarly, the Federal Ministry of Health of Ethiopia (FMOHE) developed and amended a strategy to reduce risk factors and encourage a healthy lifestyle to prevent and control noncommunicable diseases (NCDs), including BC (15). On the other hand, the population of Ethiopia is diverse, representing a wide range of lifestyles, cultures, socioeconomic statuses, and breastfeeding practices. These elements may have an impact on overall health, which may include BC risk (15).

However, to the best of our knowledge, most of the existing studies are cross-sectional, and little is known about BC determination in Ethiopia or in the study area. Therefore, this study aimed to identify determinants of BC among women attending oncology units in the Sidama Region in selected hospitals.

## 2 Materials and methods

### 2.1 Study design

A hospital-based unmatched case-control study design was employed at Hawassa City from 1 June– to 30 July 2023.

### 2.2 Study area and period

The study was conducted in the public and private health facilities of Hawassa City. The Hawassa City Administration is located in Sidama Region, Ethiopia, approximately 275 km south of Addis Ababa. The city had a total of 385,257 populations with male dominance in number. Of the total population, 89,765 were females of reproductive age.

Cities are administrative structures with dense grid populations of more than 1,500 people per km^2^. Within a city, there are small units of administrative structure called subcities. Therefore, in Hawassa City, there are eight small administrative units composed of the smallest administrative units called kebeles. Based on the above explanation, Hawassa City had eight subcities (small administrative units), and each subcity had a different number of smallest administrative units called kebeles accounting for 32 kebeles. Of these smallest administrative units, 11 were rural and 21 were urban kebeles. Hawassa City also has 8 hospitals (3 public and 5 private), 12 public health centers, and 18 health posts. The three public hospitals are Hawassa University Comprehensive Specialized Hospital (HUCSH), Adare General Hospital (AGH), and Hawela Tula Primary Hospital (HTPH) (11). According to the 2022 Sidama Regional State Health Bureau report, a flow of approximately 177 patients per month is expected. The study was performed from 1 June to 30 July 2023.

### 2.3 Population

#### 2.3.1 Source population and study population

All women who were more than 15 years old and who presented at Hawassa City health facilities were the source population. All women with confirmed BC visiting the selected health facilities for cases and all women without BC visiting the selected health facilities for other services during the study period composed the study population.

### 2.4 Inclusion and exclusion criteria

The patients were all women over the age of 15 years who had BC that had been confirmed and were receiving chemotherapy. The controls were all women over the age of 15 years who visited selected hospitals for other unrelated to cancer disease in the period of the study. However, women who were mentally incompetent or seriously ill during the course of data collection were excluded from both the case and control groups.

### 2.5 Sample size determination

Using the statistical software Epi-info version 7, the sample size for unmatched case-control studies was calculated. The following assumptions were used: 95% confidence level, 80% power, a case-to-control ratio of 1:3, a percentage of exposure among control-exposed women (i.e., the percentage of overweight and obese women without BC), and a percentage of exposure among cases (i.e., the percentage of obese women with BC).

The proportion of obese individuals among the controls was 25%, and the proportion of obese individuals among the patients was 31.9%, based on research performed in Addis Ababa, Ethiopia. Based on the above assumptions, the sample size was 272 (66 cases and 204 controls), and after adding a 10% nonresponse rate, the final sample size for the study was 300 (75 cases and 225 controls) (**Table 1**).

**Table 1:** Sociodemographic characteristics of the study participants in selected health facilities in Hawassa City, 2023.

Variables	Category	Case n (%)	Case n (%)	Total case n (%)	Pearson correlation (two tailed)	p-Value
Age in years	≤40	30 (40.0)	139 (61.8)	169 (56.3)	0.018	0.757
	40–49	18 (24.0)	33 (14.7)	51 (17.0)		
	50–59	12 (16.0)	23 (10.2)	35 (11.7)		
	≥60	15 (20.0)	30(13.3)	45 (15.0)		
Place of residence	Urban	47(62.7)	132 (58.7)	179 (59.7)	0.083	0.152
	Rural	28 (37.3)	93 (41.3)	121 (40.3)		
Educational status	No formal education	19 (25.3)	65 (28.8)	84 (28.0)	−0.035	0.548
	Primary	28 (37.3)	82 (36.4)	110 (36.6)		
	Secondary	11 (14.6)	37 (16.4)	48 (16.0)		
	College and above	17 (22.6)	41 (18.2)	58 (19.3)		
Marital status	Married	50 (66.6)	162 (72.0)	212 (70.6)	0.010	0.857
	Currently unmarred^¥^	25 (33.3)	63 (28.0)	88 (29.3)		
Occupation	Housewife	26 (34.6)	79 (35.1)	105 (35.0)	0.068	0.239
	Employee	12 (16.0)	49 (21.7)	61 (20.3)		
	Merchant	21 (28.0)	46 (20.4)	67 (22.2)		
	Farmer	11 (14.6)	34 (15.1)	45 (15.0)		
	Others*	5 (6.6)	17 (7.5)	22 (7.3)		

¥Single, divorced, and widowed. *Students and daily laborer.

### 2.6 Sampling technique and procedures

The study was performed in two hospitals, namely, Hawassa University Comprehensive (HUCSH) and Specialized Hospital and Yanet Internal Medicine Specialized Center (YIMSC), which provide chemotherapy services. The final calculated sample size was proportionally allocated to each hospital based on the number of patients who received chemotherapy services for BC treatment within the last year’s data from the health management information system (HMIS) report. Hence, 46 cases and 138 controls from HUCSH and 29 cases and 87 controls from YIMSC were included in the study. Since the cases are rare, we included all cases sequentially, but controls were included using a systematic random sampling technique. Therefore, once we included one patient, the next three controls were included in the study until the sample size was met.

### 2.7 Study variables

#### 2.7.1 Dependent variable: breast cancer

Dependent variable is breast cancer.

#### 2.7.2 Independent variables

Sociodemographic variables such as age, educational level, occupation, place of residence, and family history of cancer; behavior and/or alcohol intake, smoking status, fatty diet intake, and body mass index; reproductive variables such as age at first delivery, age at menarche, parity, breastfeeding practice, and use of hormonal contraceptives; diet and lifestyle variables such as vegetable intake, exercise, and fruit intake; and environmental and health-related variables such as pesticide contact, radiation exposure, breast trauma, and history of breast infection.

### 2.8 Data collection instruments and procedures

#### 2.8.1 Data collection instruments

Primarily, the tool was developed in English after reviewing different literature from previous studies (16–18). Then, the questionnaire was translated into the local languages Sidamagna Afoo and Amharic by an expert and translated back to English by another expert to maintain consistency.

#### 2.8.2 Data collection procedures

One day of training was given for two data collectors and one supervisor on the instrument. The interviewer administered a questionnaire supplemented with a checklist to collect data using the Kobo toolbox. Hence, during the interview, the data collector interviewed study participants about their sociodemographic, behavioral, clinical, and reproductive matters for both cases and controls. In addition, other pertinent determinant factors were identified from patients’ medical records. Pretests were performed on 5% of the sample before the actual data collection, and the necessary amendments were made.

The patient’s interview and the review of the patient’s records were used to gather information about the patient’s sociodemographic (age, residence, marital status, and level of education) and behavioral (smoking, BMI, alcohol consumption, and physical activity) BMI results. Patients were classified as underweight (<18.5), normal weight (18.5–24.9), overweight (25–29.9), or obese (>30). Reproductive (parity, OC use, menopausal status, breastfeeding practices) characteristics. Before the real data collection process, a pretest was conducted using the collected data.

### 2.9 Data quality assurance

To ensure data quality, first, the data collection tools were prepared in English, translated to Amharic, and then returned to English to ensure consistency. Appropriate training was given to the data collectors and supervisors. The training included a briefing on the general objectives of the study, approach to accessing study participants, clarity on each item in the instrument, data collection procedure, including or excluding the target data source, timeliness of data submission, data handling, and time management. Pretests were performed outside the study health institution on 5% of the sample before the actual data collection, and the necessary correction was made based on the pretest results to avoid any confusion and for better completion of the questions. Every day, the collected data were reviewed and cross-checked for completeness.

### 2.10 Data processing and analysis

The data were collected via Kobo data collection and exported to SPSS version 27 for analysis. Descriptive statistics, such as frequency and percent distribution, were used to present categorical variables. Means and standard deviations were used for continuous variables. We conducted an independent sample t test to evaluate the equality of variance and mean difference among cases and controls to exposure variables to evaluate the mean difference in exposure between cases and controls. To identify factors related to BC, a binary logistic regression analysis was performed. According to the bivariate analysis, variables with a p-value of 0.25 were candidates for multivariate analysis. To demonstrate the strength of the link, an adjusted odds ratio with a 95% confidence interval was calculated. The association of the dependent and independent variables was considered significant at a p-value of less than 0.05. The goodness of fit was checked by the Hosmer and Lemeshow test (p-value = 0.86). Moreover, multicollinearity was assessed for each variable considering variance inflation factor (VIF) values less than 10.

### 2.11 Operational definitions

BC: Women who had a confirmed BC diagnosis according to histological examination. Case: Women with BC and histologically confirmed cases (8). Controls: Women who visited gynecological OPD in selected hospitals for noncancer care. Cancer: This is a condition in which body cells multiply uncontrollably and become contagious to other parts of the body (19). BMI: Somebody’s mass divided by the square of height in meters (20). For menopausal status, women who met any of the following criteria were classified as postmenopausal: 1) had menstruation for no less than 1 year (any age) and 2) had undergone bilateral oophorectomy or estrogen deprivation therapy (21). Active smoking is a condition in which people (including former smokers) have a history of smoking within 6 months (22). Passive smoking: People who are not smokers but are exposed to tobacco smoke for more than 15 min at least 1 day per week (22).

## 3 Results

### 3.1 Sociodemographic characteristics

A total of 300 women (75 cases and 225 controls) participated in the study for a response rate of 100%. Thirty (40%) of the cases and 139 (61.78%) of the controls were aged less than 40 years with the mean ( ± SD) age of the women being 37.2 ( ± 14.8) and 36.6 ( ± 15.1) for the cases and controls, respectively. More than half of the participants [47 (62.67%) cases and 132 (58.67%) controls] were urban dwellers. Seventeen (22.67%) patients and 41 (18.22%) controls had a college education or above. Regarding occupational status, 26 (34.67%) patients and 79 (35.11%) controls were housewives (1).

### 3.2 Reproductive health-related characteristics

Of the total respondents, 34.67% (n = 26) of the patients and 22.22% (n = 50) of the controls had experienced their first menstrual period before the age of 12 years, and the mean ( ± SD) ages of the women who experienced menarche were 13.5 ( ± 2.1) and 14 ( ± 1.92) years for the cases and controls, respectively. Approximately 10 (13.33%) patients and 8.44% (n = 19) of the controls had a history of abortion. Forty-two (56%) patients and 26.22% (n = 59) of the controls were premenopausal women. More than a quarter (29.33%) of the patients and 16.44% (n = 37) of the women had irregular menstrual periods. More than half (58.67%) of the patients and 36.89% (n = 83) of the controls used oral contraceptives. In the majority of the patients, 66.6% (n = 50) and 73% (n = 162) of the controls had never breastfed their infants. One-quarter 25.33%, n = 19) of the patients and 27.11% (n = 61) of the controls had a history of breast infection (**Table 2**).

**Table 2:** Reproductive health-related characteristics of the women included in the study in selected health facilities in Hawassa City, 2023 (n = 300).

Variables	Category	Case n (%)	Control n (%)	Total Case in n (%)	Pearson correlation (two tailed)	p-Value
Menopausal status	Premenopausal	42 (56.0)	59 (26.2)	101 (33.6)	−0.040	0.714
	Menopausal	33 (44.0)	166 (73.7)	199 (66.3)		
History of abortion	Yes	10 (13.3)	19 (8.4)	29 (9.6)	0.095	0.165
	No	65 (86.6)	206 (91.5)	271 (90.3)		
Menstrual period regularity	Yes	53 (70.6)	188 (83.5)	241 (80.3)	−0.002	0.976
	No	22 (29.3)	37 (16.4)	59 (19.6)		
Age at menarche in years	≤12	26 (34.6)	50 (22.2)	76 (25.3)	0.011	0.856
	13-16	35 (46.6)	132 (58.6)	167 (55.6)		
	≥16	14 (18.6)	43 (19.1)	67 (19.0)		
Ever used an oral contraceptive	Yes	44 (58.6)	83 (36.8)	127 (42.3)	−0.152	0.008
	No	31 (41.3)	142(63.1)	173(57.6)		
Breastfeeding	Yes	50(66.6)	162 (72.0)	212 (70.6)	−0.051	0.381
	No	25 (33.3)	63 (28.0)	88 (29.3)		
Breast injury	Yes	2 (2.6)	12 (5.3)	14 (4.6)	−0.055	0.345
	No	73 (97.3)	213 (94.6)	286 (95.3)		
Breast infection	Yes	19 (25.3)	61 (27.1)	80 (26.6)	−0.017	0.764
	No	56 (74.6)	164 (72.8)	220 (73.3)		

### 3.3 Behavioral and biological-related characteristics

Among the patients, 30.67% (n = 23) and 16.00% (n = 36) of the controls had a family history of cancer. Forty percent (n = 30) of the patients and 25.33% (n = 57) of the controls were overweight and/or obese. In the majority of the patients, 77.33% (n = 58) and 70.22% (n = 158) of the controls did not participate in regular physical exercise. In the majority of the participants, 88% (n = 66) of the patients and 82.22% (n = 185) of the controls ate fewer than seven servings of fruit per week. Regarding exposure to smoking, 18.67% (n = 14) of the patients and 22.22% (n = 50) of the controls were exposed. Approximately 40% (n = 30) of the patients and 30.22% (n = 68) of the controls used packed food or drinks. Approximately 50.67% (n = 38) of the patients and 33.78% (n = 76) of the controls consumed solid oil. Among the participants, 9.33% (n = 7) of the patients and 8.8% (n = 8) of the controls had ever undergone a mammogram. Approximately 45.33% (n = 34) of the patients and 44.4% (n = 100) of the controls had practiced self-breast examinations (**Table 3**).

**Table 3:** Behavioral and biological characteristics of the study participant women in selected health facilities in Hawassa City, 2023.

Variables	Category	Case n (%)	Control n (%)	Total case in n (%)	Pearson correlation (two tailed)	p-Value
Family history of cancers	Yes	23 (30.6)	36 (16.00)	59 (19.67)	0.16	0.006
	No	52 (69.3)	189 (84.00)	241 (80.33)		
BMI	Normal	35 (46.6)	142 (63.11)	177 (59.00)	0.122	0.035
	Underweight	10 (13.3)	26 (11.56)	36 (12.00)		
	Overweight and/or Obesity	30 (40.0)	57 (25.33)	87 (29.00)		
Physical activity	Yes	17 (22.6)	67 (29.78)	84 (28.00)	0.085	0.144
	No	58 (77.3)	158 (70.22)	216 (72.00)		
Fruit intake per week	≤7	66 (88.0)	185 (82.22)	251 (83.67)	−0.141	0.014
	>7	9 (12.0)	40 (17.78)	49 (16.33)		
Exposure for smoking	Yes	14 (18.6)	50 (22.22)	64 (21.33)	−0.113	0.051
	No	61 (81.3)	175 (77.78)	236 (78.67)		
Use of packed food or drinks	Yes	30 (40.0)	68 (30.2)	98 (32.6)	0.126	0.029
	No	45 (60.0)	157 (69.7)	202 (67.3)		
Solid oil	Yes	38 (50.6)	76 (33.7)	114 (38.0)	0.05	0.0
	No	37 (49.3)	149 (66.2)	186 (62.0)		
Ever had a mammogram	Yes	7 (9.3)	18 (8.0)	25 (8.3)	0.021	0.719
	No	68 (92.0)	207 (90.6)	275 (91.6)		
Breast self-examination	Yes	34 (45.3)	100 (44.4)	134 (44.6)	−0.008	0.894
	No	41 (54.6)	125 (55.5)	166 (55.3)		

### 3.4 The mean difference in exposure between cases and controls

In this study, we conducted an independent-sample t-test to evaluate the equality of variance and mean difference among cases and controls to exposure variables. To check the similarity of variance among cases and controls, Levene’s test for equality of variances was used (i.e., equal variances, p > 0.05, and unequal variances p < 0.05). Similarly, to identify the mean difference among cases and controls, a t-test for the equality of means with respective p-values was used [p < 0.05 indicates a significant difference in the means of the two sample populations tested (cases and controls)]. Since the variance is greater than 4 for almost all of the test variables, we assumed unequal variance and used a one-sample t-test (23). The factors associated with an increased risk of BC among women were family history of cancer (p < 0.014), consumption of packed food (p < 0.03), BMI (p < 0.04), fruit consumption (p < 0.03), menopausal status (p < 0.01), and smoking status (p < 0.01) (**Table 4**).

**Table 4:** Independent-sample t-test of respondents for determinants of BC among women attending oncology units at selected health facilities of Hawassa City, Sidama Region, Ethiopia, 2023 (n = 75 cases and n = 225 controls).

Independent-sample test		Levene’s test for equality of variances		t-Test for equality of means						
		F	Sig.	t	df	Sig. (two tailed)	Mean difference	Std. error difference	95% Confidence interval of the difference	
									Lower	Upper
Place of residence	EVA	11.063	0.001	−1.435	298	0.152	−0.093	0.065	−0.221	0.035
	EVNA			−1.471	132.578	0.144	−0.093	0.063	−0.219	0.032
Family history	EVA	25.066	0.000	−2.794	298	0.006	−0.147	0.052	−0.250	−0.043
	EVNA			−2.489	106.597	0.014*	−0.147	0.059	−0.264	−0.030
Physical activity	EVA	10.532	0.001	−1.465	298	0.144	−0.089	0.061	−0.208	0.031
	EVNA			−1.540	138.901	0.126	−0.089	0.058	−0.203	0.025
Packed food	EVA	10.998	0.001	−2.197	298	0.029	−0.138	0.063	−0.261	−0.014
	EVNA			−2.108	118.628	**0**.037*	−0.138	0.065	−0.267	−0.008
BMI	EVA	2.861	0.092	−2.114	298	0.035	−0.249	0.118	−0.481	−0.017
	EVNA			−2.042	119.828	0.043*	−0.249	0.122	−0.490	−0.008
Duration of household activities	EVA	16.478	0.000	−2.067	275	0.040	−2.009	0.972	−3.922	−0.096
	EVNA			−1.698	88.855	0.093	−2.009	1.183	−4.360	0.342
Fruit consumption	EVA	22.576	0.000	2.467	298	0.014	0.102	0.041	0.021	0.184
	EVNA			2.088	100.057	0.039*	0.102	0.049	0.005	0.199
Ever used an oral contraceptive	EVA	3.642	0.057	2.659	298	0.008	0.173	0.065	0.045	0.302
	EVNA			2.615	123.390	0.010*	0.173	0.066	0.042	0.305
Menopausal status	EVA	3.642	0.057	2.659	298	0.008	0.173	0.065	0.045	0.302
	EVNA			2.615	123.390	0.010*	0.173	0.066	0.042	0.305
Passive smoker	EVA	19.012	0.000	1.959	298	0.051	0.107	0.054	0.000	0.214
	EVNA			2.188	157.178	0.030*	0.107	0.049	0.010	0.203

BMI, body mass index; EVA, equal variances assumed; EVNA, equal variances not assumed; BC, Breast Cancer.

The bold value indicating statistically significant value.

### 3.5 Factors associated with BC

According to the bivariate logistic regression analysis, age, menopausal status, use of packed foods or drinks, history of abortion, regularity of menstruation, age at menarche, body mass index (BMI), family history of cancer, use of oral contraceptives, regular physical exercise, and consumption of solid oil were independently associated with BC. However, in the multivariate logistic regression analysis, menopausal status, overweight and obesity status, use of oral contraceptives, family history of cancer, and consumption of solid oil were independently associated with BC.

The odds of having BC were almost three times greater among postmenopausal women than among their male counterparts [adjusted odds ratio (AOR) = 2.5; 95% CI: 1.18–5.2, p = 0.01]. Women with a family history of cancer were 2.33 times more likely to develop BC than women without a family history of cancer (AOR = 2.33; 95% CI: 1.12–4.82, p = 0.02). The odds of developing BC were almost three times greater among oral contraceptive users than among their counterparts (AOR = 2.74; 95% CI: 1.34–5.99, p = 0.005). Similarly, the odds of having BC were almost two times greater among overweight women than among normal-weight women (AOR = 2.29; 95% CI: 1.14–4.59, p = 0.01). Finally, women who used palm/solid oil were 2.4 times more likely to have BC than were their male counterparts (AOR = 2.36; 95% CI: 1.20–4.67, p = 0.01) (**Table 5**).

**Table 5:** Bivariate and multivariate logistic regression analysis of factors associated with BC risk among women who attended selected health facilities in Hawassa City, 2023.

Variables	Category	Case	Control	COR (95% CI)	AOR (95% CI)	p-Value
Age in years	≤40	30	139	1		
	40–49	18	33	2.52 (1.25, 5.07)	1.03 (0.43, 2.47)	0.934
	50–59	12	23	2.41 (1.08, 5.38)	1.45 (0.52, 4.04)	0.477
	≥60	15	30	2.31(1.11, 4.83)	1.66 (0.61, 4.50)	0.316
Menopausal status	Premenopausal	42	59	1	1	
	Postmenopausal	33	166	3.58 (2.07, 6.17)	2.49 (1.18, 5.23)	0.016**
History of abortion	Yes	10	19	1.67 (0.73, 3.76)	2.16 (0.79, 5.86)	0.130
	No	65	206	1	1	
Menstrual period regularity	Yes	53	188	0.47 (0.25, 0.87)	1.14 (0.51, 2.55)	0.744
	No	22	37	1		
Age at menarche in years	≤12	26	50	1.59 (0.74, 3.43)	1.81 (0.73, 4.49)	0.196
	13–16	35	132	0.81 (0.40, 1.65)	0.77 (0.31, 1.90)	0.573
	≥16	14	43	1	1	
Ever used an oral contraceptive	Yes	44	83	2.42 (1.42, 4.13)	2.74 (1.34, 5.59)	0.005***
	No	31	142	1	1	
Family Hx of cancer	Yes	23	36	2.32 (1.26, 4.25)	2.33 (1.12, 4.82)	0.023***
	No	52	189	1	1	
BMI	Normal	35	142	1	1	
	Underweight	10	26	1.56 (0.68, 3.53)	2.05 (0.77, 5.41)	0.145
	Overweight and/or obesity	30	57	2.13 (1.19, 3.80)	2.29 (1.14, 4.59)	0.020***
Physical activity	Yes	17	67	1	1	
	No	58	158	1.44 (0.78, 2.66)	1.98 (0.89, 4.38)	0.090
Fruit intake per week	≤7	66	185	1	1	
	>7	9	40	0.63 (0.29, 1.37)		
Use of packed food or drinks	Yes	30	68	1.59 (0.89, 2.64)	1.69 (0.84, 3.40)	0.136
	No	45	157	1	1	
Solid oil	Yes	38	76	2.01 (1.18, 3.42)	2.36 (1.20, 4.67)	0.013***
	No	37	149	1	1	

***Significant at a p-value <0.001 and *significant at a p-value <0.05. AOR, adjusted odds ratio; BMI, body mass index; COR, crude odds ratio.

## 4 Discussion

In this facility-based study, we evaluated the determinants of BC among women attending oncology health facilities in Hawassa City, Sidama Region, Ethiopia. Postmenopausal status (p < 0.016), BMI (p < 0.020), family history of cancer (p < 0.023), use of oral contraceptives (p < 0.02), and consumption of saturated fat (p < 0.013) were independently associated with BC.

Our study showed that menopausal status significantly increases BC risk with postmenopausal women having almost three times greater odds than premenopausal women. This finding is similar to that of a study in Addis Ababa (24). Another study from Malaysia showed that postmenopausal women had a 52% greater risk of BC (25). A meta-analysis from India comparable with this study showed that postmenopausal women have a 35% greater risk of developing BC than premenopausal women (26). A possible explanation could be that postmenopausal women face an increased risk of BC due to decreased estrogen levels, obesity, and a sedentary lifestyle, which can increase the risk of developing the disease. Thus, a systematic review and meta-analysis of evidence suggested that postmenopausal women should have regular physical activity and a healthy diet to prevent overweight and obesity to address BC (27).

Similarly, a family history of cancer significantly increases the risk of BC, and women with a history of BC are 2.3 times more likely to develop BC. This result is in line with research in Addis Ababa (8), which demonstrated that women with a family history of BC are substantially more likely to develop BC than other women. Early detection and screening could be key for BC progression. Families with a history of BC may also be more aware of the risks and symptoms leading them to seek regular screening and early detection. While this proactive approach to healthcare is important, it can create a perception of greater risk due to increased awareness and monitoring within the family.

The study revealed that oral contraceptives significantly increased BC risk with women using oral contraceptives having 2.74 times greater odds of developing BC than nonusers. This result is in line with a study conducted in Jordan and Ethiopia (28, 29). Naturally occurring estrogen and progesterone stimulate the development and growth of some cancers (e.g., cancers that express the receptor for these hormones, such as BC). Birth control pills contain synthetic versions of these female hormones. In contrast, oral contraceptives and BC were not significantly linked in a study in Ethiopia (24). The possible reason for this slight increase in risk could be related to the hormonal components of oral contraceptives. Oral contraceptives contain synthetic versions of the hormones estrogen and progestin, which are used to prevent pregnancy by suppressing ovulation and altering the cervical mucus and the lining of the uterus. Therefore, maintaining regular BC screenings and self-exams is crucial for the early detection and treatment of BC regardless of whether a woman is using oral contraceptives.

Women’s nutritional status significantly impacts BC risk with overweight or obese women having a 2.29-fold greater risk than normal-weight women. This finding is comparable to that of a study conducted in Jordan and two other studies in Ethiopia (8, 30, 31). The possible justification could be unhealthy eating patterns that are marked by high consumption of refined carbohydrates, sugar, and saturated fats (32). This increased risk can be attributed to the higher levels of estrogen produced by adipose tissue and the fact that obese women are more physically inactive, both of which can promote the growth of BC cells.

Furthermore, consuming saturated oil significantly increases BC risk. Women consuming solid oil have 2.36 times greater odds of developing BC than nonusers. This result is in line with a study performed in the USA showing that eating saturated fat increases the risk of developing BC (33). The possible reason could be that saturated fatty acids increase low-density lipoprotein cholesterol, obesity, and free radicals, which increase the risk of BC (34). A meta-analysis revealed that a high-fat diet is a risk factor for BC (35). Another systematic review showed that an unhealthy high-fat diet may contribute to obesity and affect BC (36).

The strengths of this study included the use of an observational study design with a 1:3 case-to-control ratio, which enhances its ability to identify BC determinants and its validity due to its multicenter nature. The possible study limitations include the small number of patients in the health facility, recall bias, and over- or underreporting of self-reported data on dietary consumption and physical activity.

## 5 Conclusion

BC is a public health problem both globally and in Ethiopia. This study aimed to identify the determinants of BC. Therefore, postmenopausal status, BMI, family history of cancer, being overweight, using oral contraceptives, and consuming solid oil were identified as risk factors for BC. Therefore, women should adopt healthier lifestyles through healthy nutrition and regular physical exercise, which might contribute to reducing the risk of developing BC in women. In addition, early detection and regular screening are proactive approaches for identifying BC.

## Data availability statement

The original contributions presented in the study are included in the article/supplementary material. Further inquiries can be directed to the corresponding authors.

## Ethics statement

Ethical clearance was obtained from the Ethical Review Committee of Yanet-Liyana College of Health Science (with Ref # of LHC/YLCHS/OGL/981/15 and Date: 29/05/2023 GC). The studies were conducted in accordance with the local legislation and institutional requirements. The participants provided their written informed consent to participate in this study.

## Author contributions

SK: Conceptualization, Data curation, Formal analysis, Funding acquisition, Investigation, Methodology, Project administration, Resources, Software, Supervision, Writing – original draft, Writing – review & editing. TA: Conceptualization, Data curation, Formal analysis, Investigation, Methodology, Project administration, Software, Supervision, Writing – original draft, Writing – review & editing. AM: Data curation, Formal analysis, Investigation, Methodology, Software, Validation, Visualization, Writing – original draft, Writing – review & editing.
